# Subacute exposure to di-isononyl phthalate alters the morphology, endocrine function, and immune system in the colon of adult female mice

**DOI:** 10.1038/s41598-020-75882-0

**Published:** 2020-11-02

**Authors:** Karen Chiu, Shah Tauseef Bashir, Romana A. Nowak, Wenyan Mei, Jodi A. Flaws

**Affiliations:** 1 Division of Nutritional Sciences, College of Agricultural, Consumer, and Environmental Sciences, University of Illinois at Urbana-Champaign, Urbana, IL USA; 2 Department of Comparative Biosciences, College of Veterinary Medicine, University of Illinois at Urbana-Champaign, 2001 S. Lincoln Avenue, Urbana, IL 61802 USA; 3 Department of Molecular and Integrative Physiology, College of Liberal Arts and Sciences, University of Illinois, Urbana, IL USA; 4 Department of Animal Sciences, College of Agricultural, Consumer and Environmental Sciences, University of Illinois, Urbana, IL USA; 5 Carl R. Woese Institute for Genomic Biology, University of Illinois at Urbana-Champaign, Urbana, IL USA

**Keywords:** Environmental sciences, Diseases, Endocrinology, Gastroenterology, Pathogenesis, Signs and symptoms

## Abstract

Di-isononyl phthalate (DiNP), a common plasticizer used in polyvinyl chloride products, exhibits endocrine-disrupting capabilities. It is also toxic to the brain, reproductive system, liver, and kidney. However, little is known about how DiNP impacts the gastrointestinal tract (GIT). It is crucial to understand how DiNP exposure affects the GIT because humans are primarily exposed to DiNP through the GIT. Thus, this study tested the hypothesis that subacute exposure to DiNP dysregulates cellular, endocrine, and immunological aspects in the colon of adult female mice. To test this hypothesis, adult female mice were dosed with vehicle control or DiNP doses ranging from 0.02 to 200 mg/kg for 10–14 days. After the treatment period, mice were euthanized during diestrus, and colon tissue samples were subjected to morphological, biochemical, and hormone assays. DiNP exposure significantly increased histological damage in the colon compared to control. Exposure to DiNP also significantly decreased sICAM-1 levels, increased *Tnf* expression, decreased a cell cycle regulator (*Ccnb1*), and increased apoptotic factors (*Aifm1 *and *Bcl2l10*) in the colon compared to control. Colon-extracted lipids revealed that DiNP exposure significantly decreased estradiol levels compared to control. Collectively, these data indicate that subacute exposure to DiNP alters colon morphology and physiology in adult female mice.

## Introduction

Phthalates are a large class of organic chemicals that are synthetically created from petroleum and are known to disrupt the endocrine system^1^. Phthalates were introduced in the 1920s, and they quickly replaced earlier forms of volatile and odorous plasticizers, such as camphor^1,2^. As phthalate production increased in the 1930s, so did their uses as plasticizers, stabilizers, or solvents applied to polyvinyl chloride, personal care products, detergents, medication, medical equipment, adhesives, food packaging and wraps, and pesticides^2,3^. Typically, high molecular-weight phthalates (i.e., di(2-ethylhexyl) phthalate [DEHP] and di-isononyl phthalate [DiNP]) are used as plasticizers for polyvinyl chloride. In contrast, low molecular-weight phthalates (i.e., dibutyl phthalate [DBP], diethyl phthalate [DEP], and dimethyl phthalate [DMP]) are used in personal care products to stabilize fragrances and colors^4,5^. The ubiquitous uses of phthalates and their non-covalent binding properties to materials allow phthalates to leach into the environment. They can then end up in the human body through ingestion, inhalation, and dermal absorption. However, the primary route of exposure to phthalates is through ingestion because phthalates are easily leached from the packaging materials into foods.

In this study, we focused on DiNP, which is a high-molecular-weight phthalate commonly used as a plasticizer in polyvinyl chloride products and used in materials for construction, electrical insulation, toy manufacturing, textiles (vinyl clothing or artificial leather), and other consumer products. We chose to study DiNP because studies have reported toxic effects on the male and female reproductive system, liver, and kidneys^6–9^. Furthermore, its metabolites are found in human samples, including urine, sweat, and blood^10^. Although DiNP is toxic to many organs in the body, the effects of DiNP on the gastrointestinal tract (GIT) have been overlooked, even though the GIT is often the initial site of contact in humans. Interest in DiNP has been increasing due to its structural and functional similarities to DEHP and regulations limiting the use of DEHP in certain products (i.e., children’s toys and baby bottles), leading to increased replacement of DEHP with DiNP^11–13^. It is known that the gut is a significant site of DiNP metabolism because intestinal esterases can metabolize phthalates extensively^14,15^, but whether phthalates, specifically DiNP, are toxic to the gut required further investigation.

The GIT is the largest endocrine organ in the body, and it is capable of producing more than 30 gut hormones and various bioactive peptides that serve as general intercellular messengers and regulators in the GIT^16^. Gastrointestinal hormones are produced from specialized cells called enteroendocrine cells. The enteroendocrine cells are differentiated from intestinal stem cells located in the intestinal crypts. Most of these gut hormones coming from enteroendocrine cells regulate intestinal and pancreatic function, including digestion, absorption, secretion, and motility. Gut hormones can also pass through the portal circulation, enter systemic circulation, and play a role in other physiological processes like inflammation^17^. Sex-steroid hormones, such as estradiol, are also synthesized in the gut^18^. The gut contains gut-associated lymphoid tissues (GALTs) that play an essential role in immune function^19^. Example of GALTs include mesenteric lymph nodes and Peyer’s patches, which are organized lymphoid aggregates in the intestine. Aromatase or CYP19A1 is the enzyme that converts testosterone into estradiol in the high endothelial venules of the Peyer’s patch and mesenteric lymph nodes^18^. Estradiol also plays a critical role in the immune system. Immune cells have estrogen receptors (ESRs) to help regulate immune response. Depending on the ESR isoform, the interaction between estradiol and ESR1 or ESR2 can help with immune cell proliferation or differentiation; however, this depends on the target immune cell as well as its conditions^20^. Estradiol production primarily from the Peyer’s patches and mesenteric lymph nodes can help regulate leukocyte proliferation in the gut^18^, modulate ion secretion in the GIT, maintain fluid balance, and play a role in the incidence of various gastrointestinal diseases, including peptic and duodenal ulcers^21^. Overall, gastrointestinal production of estradiol has essential roles in gastrointestinal homeostasis and immune responses.

Phthalates are classified as endocrine-disrupting chemicals (EDCs), which are exogenous chemicals that disrupt the endocrine system by binding to a receptor, mimicking hormones, or interfering with the production and regulation of hormones and their receptors. EDCs, including phthalates, have the potential to disrupt the gastrointestinal endocrine system by these similar mechanisms. In particular, DEHP and DiNP exposures have been known to alter the levels of sex-steroid hormones, such as estradiol and testosterone in the ovaries and testes^9^. Although several phthalates have been shown to disrupt the reproductive endocrine system, the effect of phthalates including DiNP on the intestinal endocrine system is unknown.

Besides being the largest endocrine organ, the gut is also the largest immune organ in the mammalian body as it houses 70–80% of the immune cells^22^. The GIT contains hemopoietic (i.e., classical immune cells—macrophages, monocytes, dendritic cells, T- and B-lymphocytes) and non-hemopoietic (i.e., intestinal epithelial cells) stem cells that support the immune system^22^. For example, different cell lineages of intestinal epithelial cells work together as a barrier against pathogens: goblet cells secrete mucus to trap pathogens and Paneth cells generate anti-microbial peptides and immunomodulating proteins to aid the immune system. Beyond GI cells that aid the immune system, tight junctions (i.e., zona occluden [ZO], occludin [OCLN], and claudin [CLDN]) in between or within intestinal epithelial cells (IECs) increase protection against external antigens^23^. In the intestines, OCLN and CLDN are transmembrane proteins that exist between IECs, whereas ZO proteins are located in the cytoplasm of IECs. Dysfunction in tight junctions may lead to local or systemic inflammation and other diseases^24^. Exposure to environmental chemicals can affect immune responses locally and systemically^25^. Although some evidence indicates that DiNP causes inflammatory responses in human monocyte cells as well as in the dermis in mice^26–28^, little is known about whether DiNP exposure alters the colonic immune microenvironment.

The cell cycle is important in all cells, including IECs. IECs have a high turnover rate of 4–5 days to help maintain an impermeable barrier to gut microbiota and luminal contents. Apical extrusion or shedding of IECs indicate they are terminated. However, a highly inflammatory milieu rich in TNF-$\alpha$ can compromise the intestinal epithelial barrier, further exacerbating inflammatory and apoptotic conditions. In vitro studies have shown that high-molecular-weight phthalates including DEHP alter the expression of genes involved in cell cycle and apoptotic pathways in human leiomyoma cells^29^, ovarian antral follicles^30^, and spermatocytes^29,31^. Disruptions in the cell cycle can either lead to abnormal cell proliferation and/or apoptosis, and this may depend on the cell type. Although apoptosis and dysregulation of the cell cycle has been observed in the male^31,32^ and female reproductive tracts^30,33^, the impact of DiNP exposure and cell cycle in the colon is unknown.

This study was designed to investigate the impacts of subacute exposure to DiNP during adulthood on the colon of female mice. Few studies have examined the impact of any phthalates on the gut, and even fewer studies have investigated the impact of DiNP on the gut. So far, only one other study has investigated the impact of DiNP on the gut, and this study reported that DiNP exposure at 380 mg/kg/day for 30 days resulted in villous atrophy in the small intestine during gestation and lactation of female rats^34^. Overall, data on how DiNP affects the gut are still scarce. Thus, this study tested the hypothesis that subacute exposure to DiNP alters the morphology, immune system, and endocrine function of the colon.

## Materials and methods

### Chemicals

DiNP, purchased from Sigma-Aldrich (St. Louis, Missouri), and corn oil, purchased from MP Biomedicals (Solon, Ohio), were the chemicals used in the dosing experiment. The DiNP doses used in this study included the following: 0, 0.02, 0.2, 2, 20, and 200 mg/kg. The highest dose (200 mg/kg DiNP stock solution) was made by diluting concentrated DiNP (as purchased from Sigma-Aldrich, CAS Number 28553-12-0) in corn oil. While creating the 200 mg/kg dose, the density of DiNP (0.972 g/mL at 25 °C) was factored into the equation. The other DiNP doses (0.02, 0.2, 2, and 20 mg/kg) were created from 200 mg/kg DiNP through serial dilutions. Corn oil was used as vehicle control.

The doses of DiNP were selected to mimic environmentally relevant exposures or typical toxicological doses that could be compared to other studies that used higher doses. For example, 0.02 mg/kg/day was to used reflect occupational exposure^35^, whereas 0.2 mg/kg/day was selected to mimic exposure in children aged 0–18 months who commonly mouth plastic toys containing DiNP^36^. The other three doses (2, 20, and 200 mg/kg/day) were selected because they are typical ranges used in traditional toxicology studies on DiNP^9,27,37,38^.

### Experimental animals

This experiment was performed in an AAALAC (Association for Assessment and Accreditation of Laboratory Animal Care)-approved animal facility. Specifically, this toxicological study was conducted in the Veterinary Medicine Basic Science Building (University of Illinois at Urbana-Champaign). Female outbred CD-1 mice (approximately 2 months old) were purchased from Charles River (Wilmington, MA), group-housed (3 mice per cage), and were allowed to acclimate to the new facility for 7 days. The facility that housed these mice kept ambient temperatures at 21.1 ± 2.2 °C, humidity at 50 ± 20%, and light:dark cycles for 12 h each cycle. Mice were also given Tekland Rodent Diet (8604) and reverse-osmosis-treated water ad libitum. All animal procedures were approved by the University of Illinois Institutional Animal Care and Use Committee or Illinois IACUC (Protocol No.: 20034 and 19110).

### Experimental design

**Figure 1:**
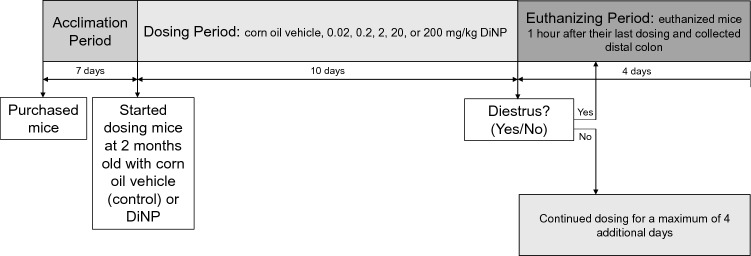
Experimental timeline shows that adult female CD-1 mice were acclimated to the animal facilities for 1 week, and then dosed with corn oil control or varying doses of DiNP (0.02 – 200 mg/kg/day) for 10 to 14 days. Mice were euthanized during diestrus one hour after their last dosing.

Two-month-old female mice (N = 36 mice total; 6 mice per treatment group) were orally dosed by gently pipetting corn oil vehicle (control) or DiNP (0.02, 0.2, 2, 20, or 200 mg/kg) into the mouth. Dosing occurred once a day in the morning for at least 10 days, and the mice were euthanized one hour after their last dosing. Estrous cycles in female mice last 4–5 days^39^ and were monitored by daily vaginal smears. Mice were euthanized by CO_2_ asphyxiation and cervical dislocation during diestrus. Mice were continually dosed with either corn oil or DiNP until they were in diestrus, which is why some mice were dosed for more than 10 days. All mice were euthanized by day 14 of the experimental timeline. See Fig. 1 for a depiction of the experimental timeline. All methods were carried out in accordance with relevant guidelines and regulations.

### Tissue collection

Colons were trimmed to remove mesenteric tissues and flushed with cold, sterile 1X PBS to remove colonic contents before weighing the mass and measuring the length of the colon. Distal colons were cut into pieces approximately 0.5 cm long and placed into sterile Eppendorf tubes. The Eppendorf tubes were then snap-frozen in liquid nitrogen and stored in − 80 °C for RNA, lipid, and cytokine extractions. Colon weight-to-length ratio was calculated by taking the weight (mg) divided the length of the colon (cm).

### Histology

Distal colons were fixed in 10% formalin (Macron Fine Chemicals, Center Valley, PA) for 24 h at 4 °C. After 24 h, the 10% formalin was replaced with 70% ethanol at 4 °C until further tissue processing. After tissue processing, formalin-fixed paraffin-embedded (FFPE) colonic tissues were sectioned at 7 µm thickness using a microtome (Microm HM 310) and mounted onto glass slides (Surgipath X-Tra Microscope Slides, Leica). Sectioned tissues were stained with Richard-Allan Scientific hemotoxylin (ThermoScientific, Kalamazoo, MI) and Richard-Allan Scientific eosin with phloxin (ThermoScientific, Kalamazoo, MI). Slides were scanned with the Hamamatsu NanoZoomer 2.0 HT (Model #C9600-12) using NDP.scan 3.2.15, and images were evaluated with NDP.view.2 software.

Colon sections were randomized and graded without knowledge of treatment group on the following attributes: enterocyte sloughing, focal or cellular inflammation, edema, crypt damage, and aberrant colon walls. The scoring system used for grading histological sections in the colon is summarized in Table 1. In detail, the attributes listed above were graded on a scale of 0 to 3, with 0 being normal, 1 being minimal or mild, 2 being moderate, and 3 being severe. Several sections (2–3 sections) were examined and graded for each colon. The grades for each characteristic (enterocyte sloughing, focal or cellular inflammation, edema, crypt damage, and aberrant colon walls) were summed to give each tissue a total score. Then, the average was taken for control and treatment groups.

**Table 1:** The scoring system used for analyzing histopathology in the colon of adult CD-1 female mice.

Item	Description of Score	
Enterocyte sloughing	0	Colonocytes are lifted from < 5% of villus tips of the luminal surface of the colon
	1	Colonocytes are lifted from 5 to 25% of villus tips of the luminal surface of the colon
	2	Colonocytes are lifted from 26 to 50% of villus tips of the luminal surface of the colon
	3	Colonocytes are lifted from > 50% of villus tips of the luminal surface of the colon
Celluar infiltration	0	No leukocyte infiltration observed in the lamina propria
	1	1–5 small aggregates near laminal surface or 10% lymphocyte infiltration
	2	1–5 small aggregates in superficial portions of the lamina propria or < 25% lymphocyte infiltration
	3	Aggregates in villi and lamina propria or > 50% lymphocyte infiltration
Edema	0	No edema
	1	Mild expansion or spreading of the cells or < 10% edema
	2	Moderate expansion or spreading of the cells or 11–25% edema
	3	Severe expansion or spreading of the cells or > 50% edema
Aberrant crypts	0	None
	1	Few distortion or 1–2 sites of aberrant crypts
	2	Mild to medium distortion or 3–5 sites of aberrant crypts
	3	Severe distortion or > 5 sites of aberrant crypts
Aberrant colon walls	0	Normal
	1	The structure of the colon wall is integrated
	2	Colon wall thickening with no ulceration
	3	Colon wall thickening invading into muscular layer with ulceration

### RNA extraction and gene expression analysis

Frozen tissues were used for RNA extraction using the RNeasy Micro Kit by Qiagen (Qiagen, Inc., Valencia, CA). RNA extraction was carried out according to the manufacturer’s instructions. Once RNA was extracted, samples were eluted in RNase-free water. RNA concentration was quantified at λ = 260 nm using NanoDrop (NanoDrop ND-1000, ThermoScientific, Waltham, MA) and then stored at − 80 °C until complementary DNA (cDNA) synthesis.

For cDNA synthesis, RNA (100 ng) was reverse transcribed into DNA using iScript Reverse Transcriptase (Bio-Rad Laboratories, Inc., Hercules, CA) according to the manufacturer’s instructions. Each qPCR reaction was done in duplicate. Each replicate contained 2 µL of cDNA, forward and reverse primers (5 pmol) for select genes, SsoFastEvaGreen Supermix, and nuclease-free water to give a final reaction volume of 10 µL. Real-time quantitative polymerase chain reactions (RT-qPCR) were carried out using the CFX96 Real-Time Detection System (Bio-Rad Laboratories) and CFX Manager Software.

All qPCR primers used for this study are listed in Table 2. Briefly, reactions were performed for interferon gamma (*Ifng*), tumor necrosis factor (*Tnf*), interleukin 4 (*Il4*), interleukin 5 (*Il5*), interleukin 6 (*Il6*), interleukin 13 (*Il13*), interleukin 17A (*Il17a*), zonula occludens-1 (*Zo-1*), zonula occludens-2 (*Zo-2*), zonula occludens-3 (*Zo-3*), occludin (*Ocln*), claudin 1 (*Cldn1*), claudin 4 (*Cldn4*), B-cell lymphoma 2 (*Bcl2*), apoptosis-inducing factor 1 (*Aimf1*), B-cell lymphoma 2 like 10 (*Bcl2l10*), cyclin B1 (*Ccnb1*), cyclin D2 (*Ccnd2*), cyclin A2 (*Ccna2*), cyclin E1 (*Ccne1*), cyclin dependent kinase inhibitor (*Cdkn1a*), cyclin dependent kinase 4 (*Cdk4*), and Kiel 67 (*Ki67*). The expression data were normalized to corresponding values for $\beta$-*Actin*, the housekeeping gene. Finally, relative fold changes were calculated using the Pfaffl method and normalized to the control group.

**Table 2:** Primer sequences of genes assessed in the colon tissues of female CD-1 mice using real-time quantitative PCR analysis.

Target gene	Sense primer (5′ → 3′)	Antisense primer (5′ → 3′)	Full name
**Inflammation**			
*Ifng*	TCATGGCTGTTTCTGGCTGT	CACCATCCTTTTGCCAGTTCC	Interferon gamma
*Tnf*	AGCCCACGTCGTAGCAAAC	TTTGAGATCCATGCCGTTGG	Tumor necrosis factor
*Il4*	CTCGAATGTACCAGGAGCCA	GTGGTGTTCTTCGTTGCTGT	Interleukin 4
*Il5*	TGACTCTCAGCTGTGTCTGG	AAGCCTCATCGTCTCATTGCT	Interleukin 5
*Il6*	ACAAAGCCAGAGTCCTTCAGAG	AGAGCATTGGAAATTGGGGT	Interleukin 6
*Il13*	CTCTTGCTTGCCTTGGTGGT	CTCCATACCATGCTGCCGTT	Interleukin 13
*Il17a*	GCCCTCAGACTACCTCAACC	CAGCTTTCCCTCCGCATTGA	Interleukin 17A
**Gut integrity**			
*Zo-1*	ATGTTTATGCGGACGGTGG	TTTCCTCCATTGCTGTGCTCTTA	Zonula occludens-1
*Zo-2*	GCAGCTTGTAGTTCTGAGCCG	TACTGCTCCCATATCACCTCCT	Zonula occludens-2
*Zo-3*	AAGTGGGGGCTGATTGTTTCCA	AGTGTGGCTGTGTGTTGTTCC	Zonula occludens-3
*Ocln*	GGTCCTCCTGGCTCAGTTG	AAGATAAGCGAACCTTGGCGG	Occludin
*Cldn1*	TCTACGAGGGACTGTGGATGT	ATTACCATCAAGGCTCGGGT	Claudin 1
*Cldn4*	AGCAAACGTCCACTGTCCTT	GGGGCGTAATGGCAAGAGTA	Claudin 4
**Cell regulators/proliferation/apoptosis**			
*Bcl2*	ATGCCTTTGTGGAACTATATGGC	GGTATGCACCCAGAGTGATGC	B-cell lymphoma 2
*Aifm1*	AGGACGGTGAGCAACATGAA	GTTCTATCCACCCATCCCGC	Apoptosis-inducing factor 1
*Bcl2l10*	CGCTACACACACTGACTGGA	CTTTAGGATCCCCTGCCCTG	B-cell lymphoma 2 like 10
*Ccnb1*	TGCATTCTCTCAGTGCCCTCCACA	AGACAGGAGTGGCGCCTTGGT	Cyclin B1
*Ccnd2*	CCTTTGACGCAGGCTCCCTTCT	ACCCTGGTGCACGCATGCAAA	Cyclin D2
*Ccna2*	GCTCTACTGCCCGGAGGCTGA	TGGCCTACATGTCCTCTGGGGAA	Cyclin A2
*Ccne1*	GGTGTCCTCGCTGCTTCTGCTT	CCGGATAACCATGGCGAACGGA	Cyclin E1
*Cdkn1a*	TTAGGCAGCTCCAGTGGCAACC	ACCCCCACCACCACACACCATA	Cyclin dependent kinase inhibitor 1A
*Cdk4*	AGAAACCCTCGCTGAAGCGGCA	TGGGGGTGAACCTCGTAAGGAGA	Cyclin dependent kinase 4
*Ki67*	GCTCACCTGGTCACCATCAA	ACTACAGGCAGCTGGATACG	Kiel 67
**Housekeeping genes**			
$\beta$*-Actin*	GGGCACAGTGTGGGTGAC	CTGGCACCACACCTTCTAC	Actin, beta

### Lipid extractions and hormone measurements

Before lipid extraction, colon weights were measured so that hormone levels could be normalized to colon weight. Lipids were extracted from the colon to determine concentrations of estradiol and testosterone. To do this, a mixture of PBS (1x):HCl (0.1 N) (5:2 v/v) was added to each sample and then homogenized. Lipids were extracted with ethyl acetate:isopropanol (1:1 v/v) and then separated with PBS:ethyl acetate (3:2 v/v) mixture. The ethyl acetate phase was collected and evaporated using Speedvac at 30 °C for 2–3 h. Lipid samples were stored at − 80 °C until hormone measurements.

Testosterone and estradiol enzyme-linked immunosorbent assay (ELISA) kits were purchased from DRG and used to measure testosterone and estradiol levels in the colon, respectively. Lypocheck (Bio-Rad Laboratories) was used as a control for the DRG ELISA kits. Testosterone and estradiol assays were carried out according to the manufacturer’s protocol. ELISA plate absorbance values were read at 450 nm with the Multiskan Ascent microtiter plate reader (Thermo Electron Corporation).

### Cytokine extraction and measurements

Frozen tissues stored in − 80 °C were weighed before cytokine extraction. Colon tissues were resuspended in T-PER Tissue Protein Extraction Reagent (ThermoFisher, Rockford, IL) and antifoam SE-15 (Millipore Sigma, St. Louis, MO). Then, tissues were homogenized using a Tissue-Tearor (Biospec Product, Inc, Model 985370-398). The homogenized solution was centrifuged for 10 min at 6000 rpm at 4 °C. The supernatant was recovered and stored at − 80 °C. A sample of the supernatant was also used to determine the protein concentration using Pierce BCA Protein Assay Kit (ThermoFisher Scientific, Rockford, IL). After cytokine extraction from the colon tissues, the samples were subjected to the mouse sICAM-1/CD54 Quantikine ELISA kit (R&D Systems, Minneapolis, MN) and the mouse TNF alpha uncoated ELISA kit (Invitrogen, Waltham, MA). ELISA protocols were followed according to manufacturer’s instructions. The sICAM ELISA assay was read with the Multiskan Ascent microtiter plate reader (Thermo Electron Corporation), and the TNF-$\alpha$ ELISA assay was read with the MQX200 UQuant microplate reader (BioTek).

### Statistical analysis

The data presented in this study were analyzed using SPSS Statistics software (SPSS Inc., Chicago, IL) and expressed as means $\pm$ standard error of the means (SEM). Data were assessed for normality using the Shapiro–Wilk test. Data that were normally distributed and that met the assumption for homogeneity of variance were analyzed using one-way analysis of variance (ANOVA). If the ANOVA test reported *p* < 0.05, we conducted post hoc analysis using Dunnett’s 2-sided test. The following data were analyzed using ANOVA: colon length, colon weight, colon weight-to-length ratio, estradiol levels, tight junctions (*Cldn-1*, *Zo-1*, *Zo-2*, and *Zo-3*), inflammation (sICAM-1, *Il5*, *Il6*), and cell health (*Ki67*, *Ccna2*, *Ccnb1*, *Ccnd2*, *Ccne1*, *Cdk4*, *Aifm1*, *and Bcl2*).

Data that were not normally distributed and that did not meet homogeneity of variance were analyzed using the Mann–Whitney U test. The following data were analyzed using the Mann–Whitney U test: colon histology, testosterone levels, tight junctions (*Ocln* and *Cldn-4*), cell cycle regulators (*Cdkn1a* and *Bcl2l10*), inflammation (*Ifng*, *Tnf,* and TNF-alpha).

Statistical significance was assigned with one or two asterisks and defined as 0.01 $\le$ *p* < 0.05 or *p* < 0.01, respectively. Borderline significance (^) was defined as 0.05 $\le$ *p* < 0.10.

## Results

### Gross measurements

**Figure 2:**
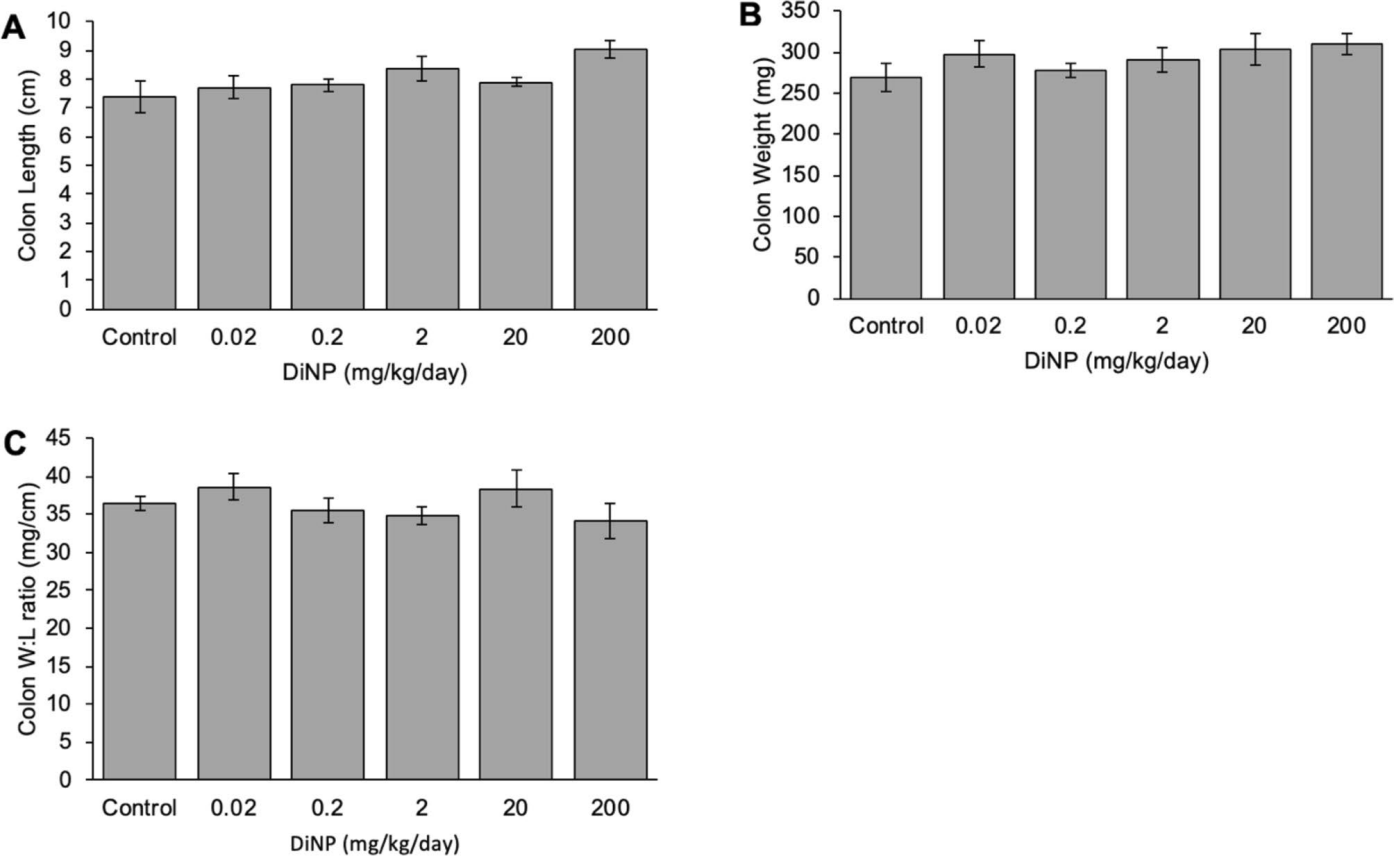
Gross measurements of the colon include length (**A**), weight (**B**), and weigh-to-length ratio (**C**). Values represent mean ± SEM, n = 6/group.

In all mice, the colon length ranged from 5.8 to 10.2 cm. DiNP exposure at all doses did not significantly affect colon length compared to control (Fig. 2A). Similarly, DiNP exposure did not markedly alter colon weight compared to control (Fig. 2B). Exposure to DiNP also did not significantly alter the colon weight-to-length ratio compared to control (Fig. 2C).

### Histopathology

**Figure 3:**
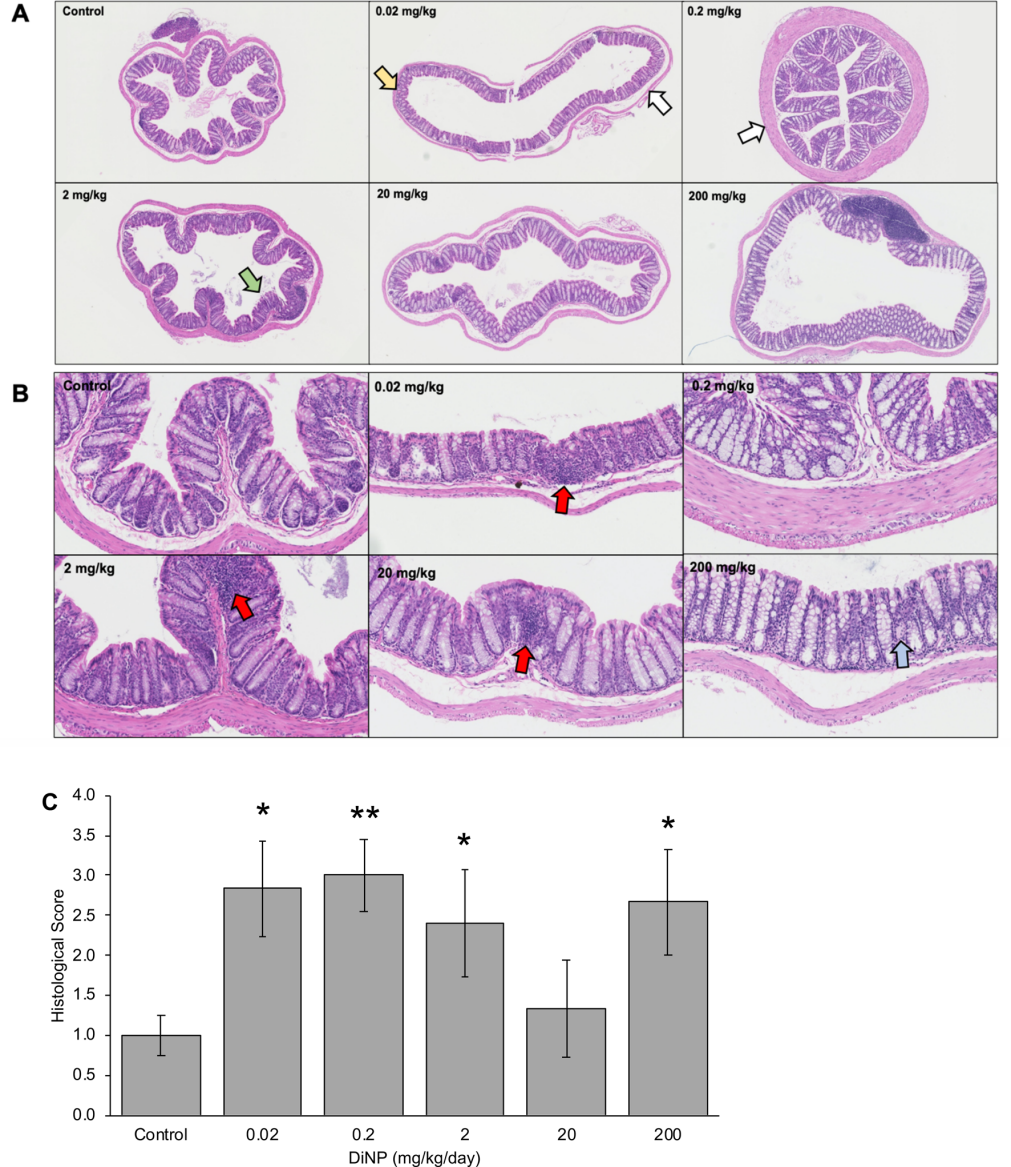
Representative histology of colon tissues treated with control or varying doses of DiNP (0.02–200 mg/kg/day) at 4X (**A**) and 20X objective (**B**). Red arrows indicate leukocyte infiltration, white arrows indicate aberrant colon walls, a yellow arrow indicates aberrant crypts, a green arrow represents enterocyte sloughing, and a blue arrow represents edema. Multiple sections of the colon were graded and given histological scores (**C**). A single asterisk (*) indicates significant differences compared to control (0.01 ≤ *p* < 0.05), and two asterisks (**) indicate very significant differences compared to control (*p* < 0.01).

Histological analysis revealed that DiNP exposure increased colonic damage compared to control (Fig. 3). Specifically, DiNP doses at 0.02, 0.2, 2, and 200 mg/kg significantly increased colonic damage compared to control (*p* < 0.05, Fig. 3C). Interestingly, the changes at the low doses of DiNP (0.02 and 0.2 mg/kg/day) were mainly due to cellular infiltration and aberrant colon walls, whereas the changes at high doses of DiNP (2 and 200 mg/kg/day) were mainly attributed to edema. Further, some enterocyte sloughing occurred in the 0.02–2 mg/kg DiNP treatment groups.

### Hormone levels

**Figure 4:**
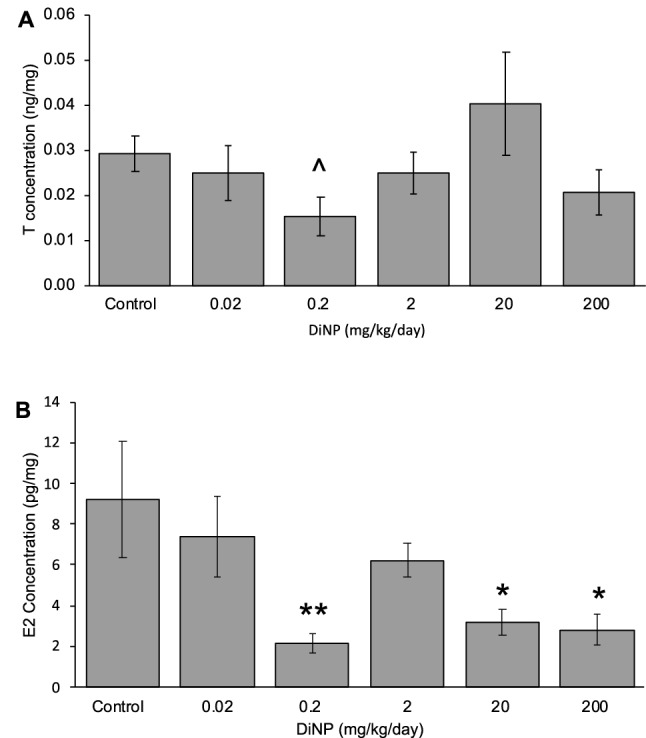
Lipids extracted from the colon for testosterone (**A**) and estradiol (**B**) measurements of adult female CD-1 mice. Values represent mean ± SEM, n = 6/group. A caret (^) symbol indicates borderline differences compared to control (*p* = 0.065). A single asterisk (*) indicates significant differences compared to control (0.01 ≤ *p* < 0.05), and two asterisks (**) indicate very significant differences compared to control (*p* < 0.01).

DiNP exposure at most doses did not significantly alter testosterone levels in the colon compared to control, but 0.2 mg/kg/day DiNP marginally decreased testosterone levels compared to control (*p* = 0.065; Fig. 4A). DiNP exposure significantly decreased estradiol concentrations at 0.2, 20, and 200 mg/kg/day compared to control in the colons (*p* < 0.05). However, DiNP exposure at 0.02 and 2 mg/kg/day did not alter estradiol levels in the colon significantly compared to control (Fig. 4B).

### Cell health

#### Cell cycle regulation

**Figure 5:**
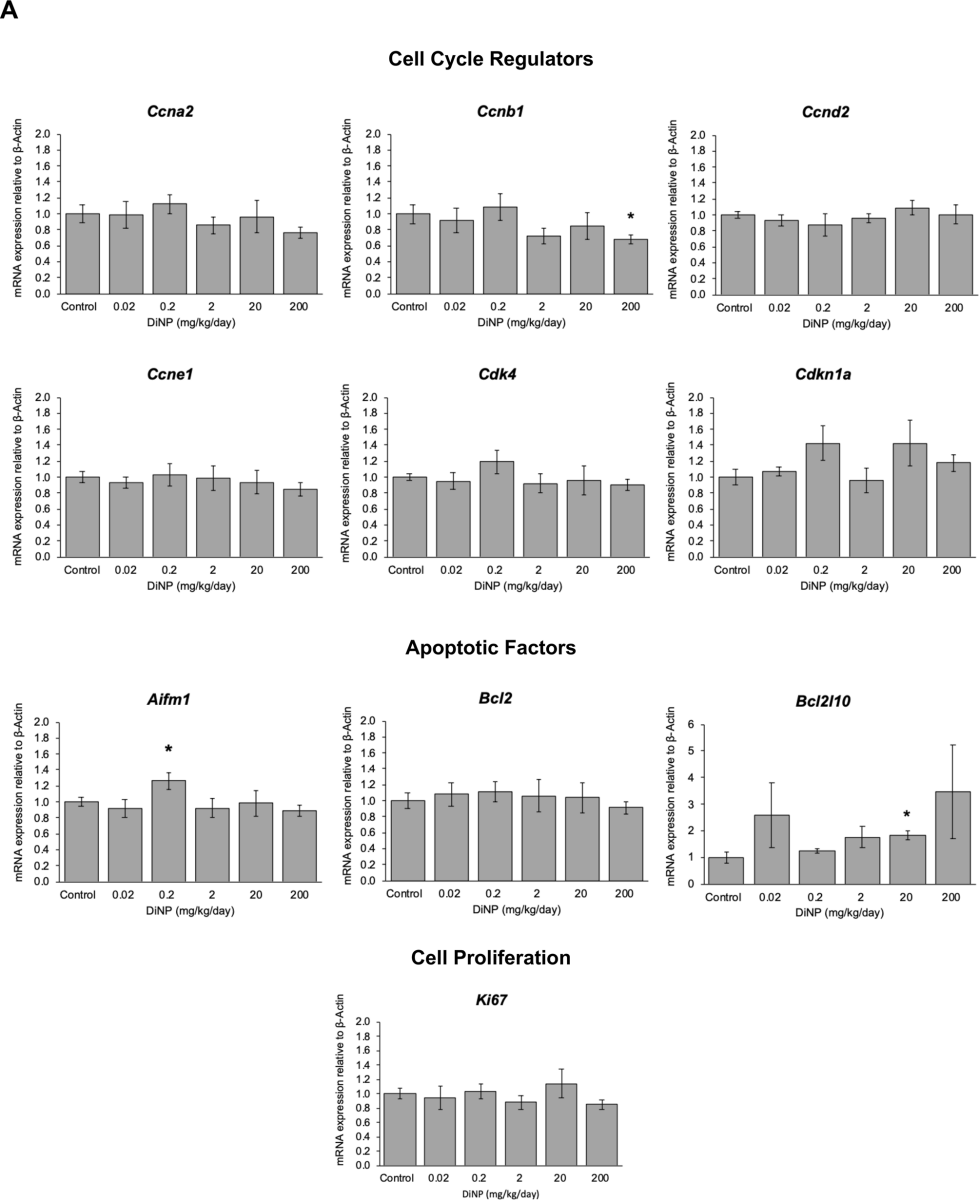

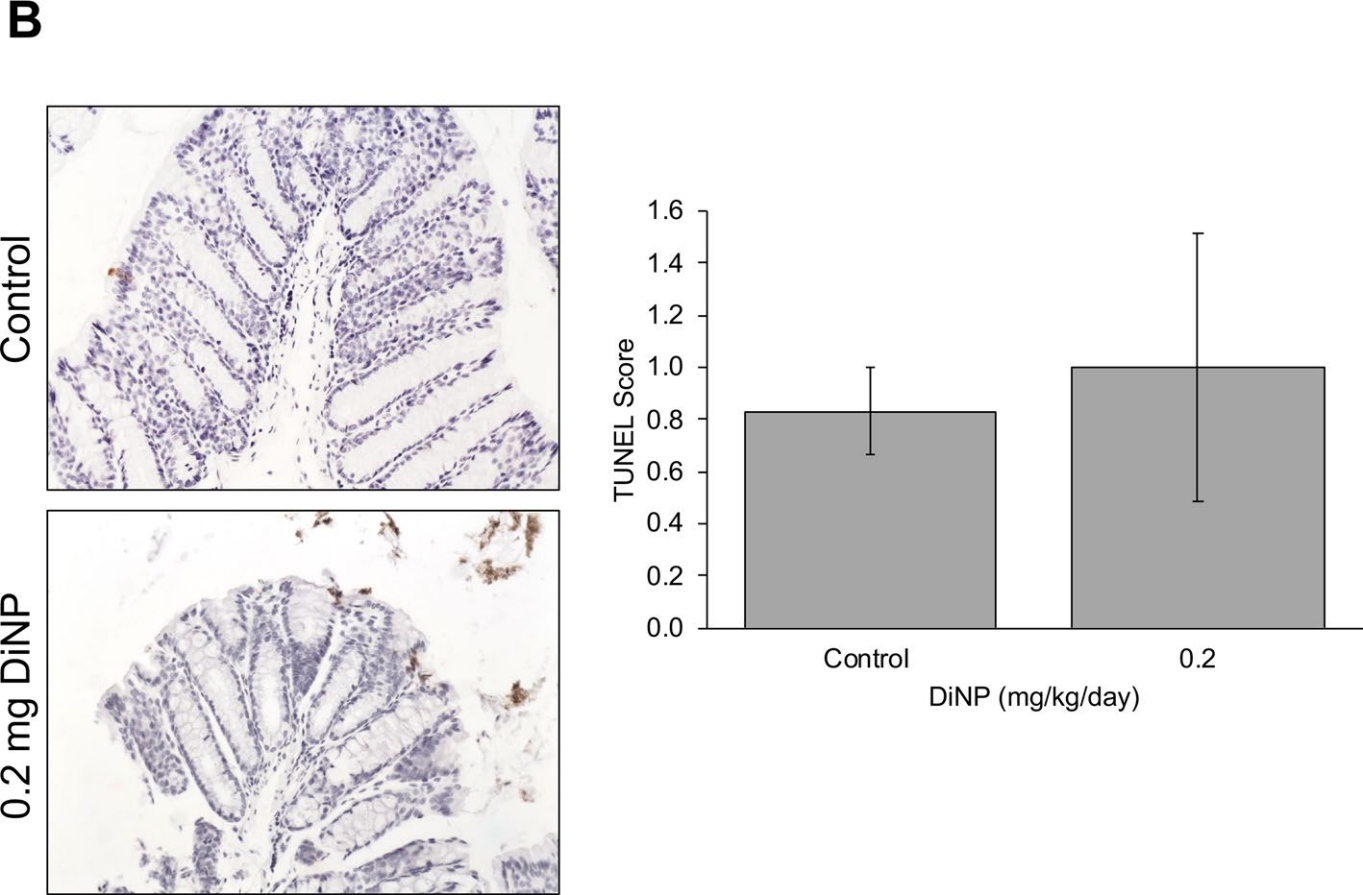
Relative expression of genes related to cell cycle regulation and cell health in terms of cell proliferation or apoptosis in colon tissue of adult female CD-1 mice exposed to corn oil control or DiNP ranging from 0.02 to 200 mg/kg/day (**A**). Values represent mean ± SEM, n = 6/group. A single asterisk (*) indicates significant differences compared to control (0.01 ≤ *p* < 0.05). TUNEL staining and quantification (**B**) was conducted to assess for apoptosis.

Expression of cell cycle factors that promote the cell cycle such as cyclin A2 (*Ccna2*), cyclin B1 (*Ccnb1*), cyclin D2 (*Ccnd2*), cyclin E2 (*Ccne1*), and cyclin dependent kinase 4 (*Cdk4*) were examined in the colon (Fig. 5A). The highest dose of DiNP (200 mg/kg) significantly decreased *Ccnb1* expression compared to control (*p* = 0.035). However, DiNP treatment did not alter expression of the other cell cycle regulators (*Ccna2*, *Ccnd2*, *Ccne1*, and *Cdk4*) compared to control. Further, DiNP treatment did not alter expression of cyclin dependent kinase inhibitor 1a or *Cdkn1a*, a cell cycle inhibitor, compared to control (Fig. 5A).

#### Apoptosis and cell proliferation

Expression of apoptotic factors including *Aifm1* and *Bcl2l10* was also examined to determine the health of cells. DiNP exposure at an environmentally relevant dose (0.2 mg/kg) significantly increased expression of *Aifm1* compared to control (*p* < 0.05). DiNP exposure (20 mg/kg) also significantly increased *Bcl2l10* expression compared to control (*p* = 0.012). Because DiNP increased expression of two pro-apoptotic factors, *Aifm1* and *Bcl2l10*, compared to control, we conducted TUNEL staining to examine whether DiNP treatment caused the DNA fragmentation that occurs during apoptosis. Interestingly, DiNP exposure did not affect TUNEL staining compared to control (Fig. 5B).

In addition to examining apoptotic factors, cell survival factors including *Bcl2* were examined in each treatment group. DiNP exposure did not alter expression of *Bcl2* compared to control (Fig. 5A). Further, DiNP exposure did not significantly alter *Ki67* expression compared to control at any dose (Fig. 5A).

### Inflammation

**Figure 6:**
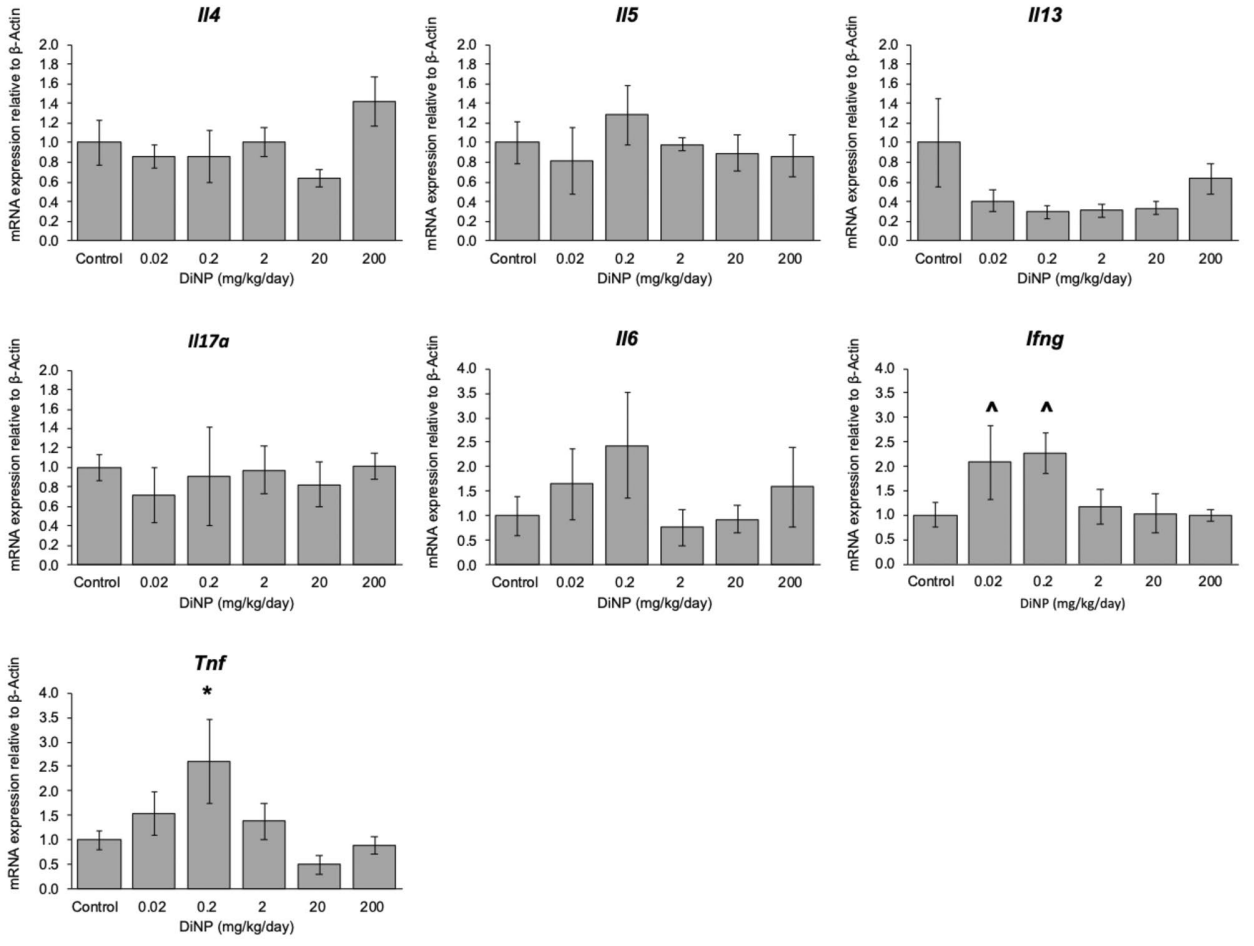
Relative expression of cytokines in colon tissue of adult female CD-1 mice exposed to corn oil control or DiNP (0.02–200 mg/kg/day). Values represent mean ± SEM, n = 6/group. A caret (^) symbol indicates borderline differences compared to control (0.05 ≤ *p* < 0.10). A single asterisk (*) indicates significant differences compared to control (0.01 ≤ *p* < 0.05).

**Figure 7:**
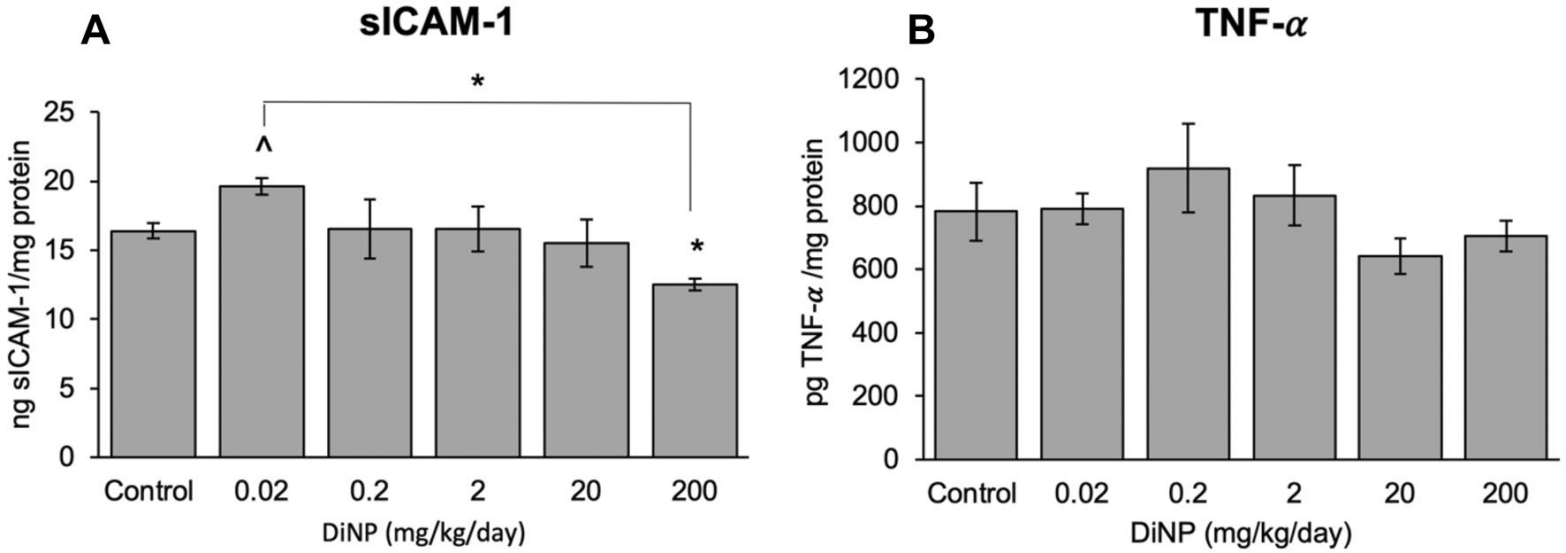
Protein levels of cytokine sICAM-1 (**A**) and TNF-$\alpha$ (**B**) in colon tissue of adult female CD-1 mice exposed to corn oil control or DiNP (0.02 – 200 mg/kg/day). Values represent mean $\pm$ SEM, n = 6/group. A caret (^) symbol indicates borderline differences compared to control (0.05 ≤ *p* < 0.10). A single asterisk (*) indicates significant differences compared to control (0.01 ≤ *p* < 0.05).

Expression of the following cytokines was measured from the distal colon: *Il4, Il5*, *Il6*, *Il13*, *Il17a*, *Tnf*, and *Ifng* (Fig. 6). DiNP exposure did not significantly alter expression of *Il4*, *Il5*, *Il6, Il13,* and *Il17a* compared to control. However, environmentally relevant doses of DiNP (0.02 and 0.2 mg/kg/day) borderline increased the expression of interferon gamma (*Ifng*). Interestingly, an environmentally relevant dose (0.2 mg/kg/day) of DiNP exposure significantly increased *Tnf* expression compared to control (*p* = 0.047, Fig. 6). Although DiNP exposure significantly increased the expression of *Tnf* compared to control, it did not alter TNF-α protein levels (Fig. 7B). Interestingly, the gene expression and protein level for this cytokine showed a similar trend and dose–response curve.

The lowest dose of DiNP exposure borderline increased sICAM-1 levels compared to control (*p* = 0.083, Fig. 7A). On the other hand, DiNP at the highest dose significantly decreased sICAM-1 compared to control (*p* = 0.016, Fig. 7A).

**Figure 8:**
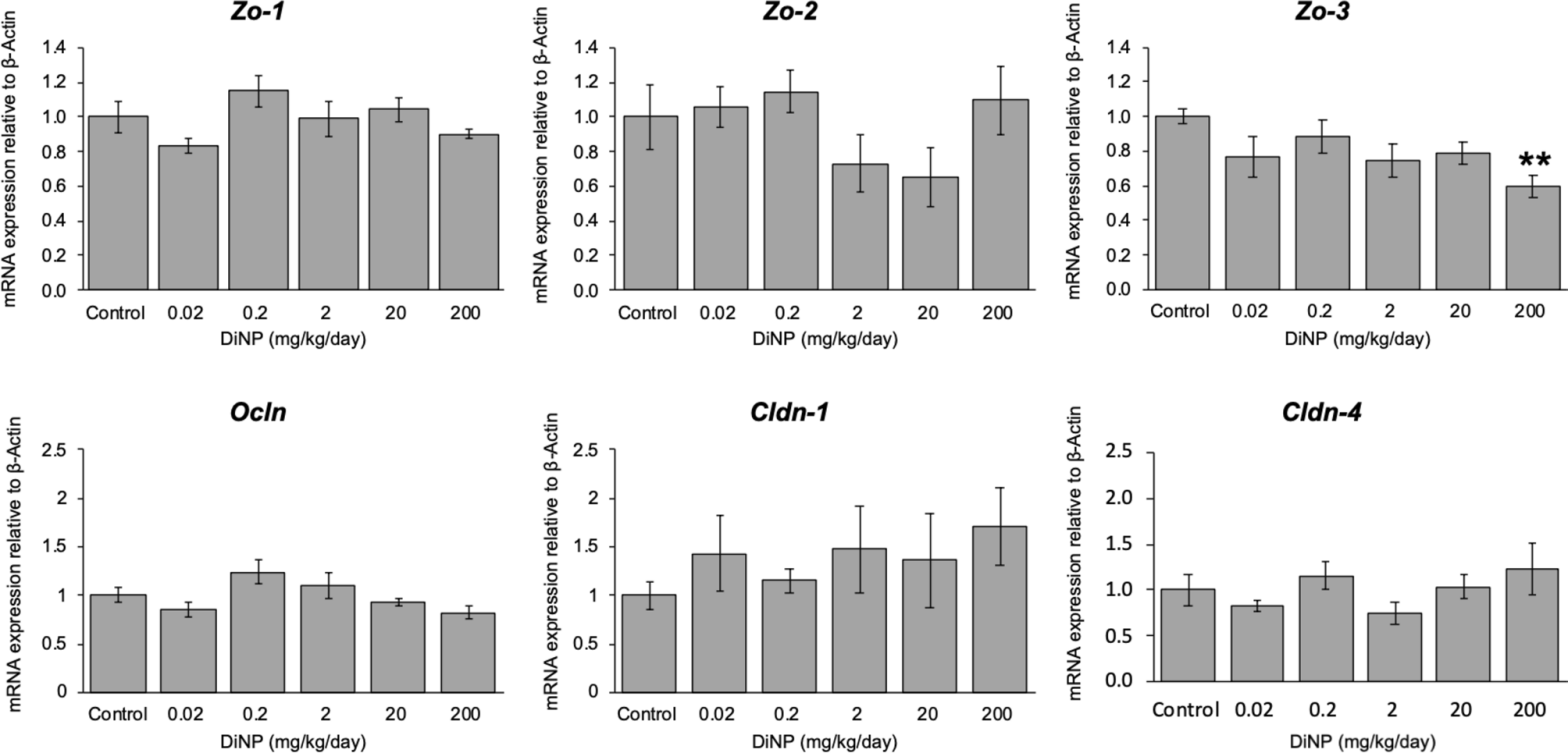
Relative expression of tight junctions in colon of adult female CD-1 mice exposed to corn oil control or DiNP (0.02–200 mg/kg/day). Values represent mean ± SEM, n = 6/group. Two asterisks (**) indicate very significant differences compared to control (*p* < 0.01).

Tight junctions play a role in mediating immune responses^40^. Thus, we examined expression of tight junction proteins such as *Zo-1*, *Zo-2*, *Zo-3*, *Cldn*1, *Cldn4*, and *Ocln* (Fig. 8). DiNP exposure did not affect the expression of *Zo-1*, *Zo-2*, *Cldn1*, *Cldn4*, and *Ocln* (Fig. 8). However, DiNP exposure at 200 mg significantly decreased expression of *Zo-3* compared to control (*p* = 0.016, Fig. 8).

## Discussion

In the present study, we showed that DiNP exposure significantly increased histological damage in the colon compared to controls. These results are consistent with previous studies that showed that subacute DBP exposure at 500 mg/kg/day significantly altered intestinal histopathology^41^. Specifically, DBP exposure significantly increased villus height and villus height/crypt depth ratio (V/C ratio) in the duodenum, whereas DBP exposure significantly decreased villus height and V/C ratio in the jejunum compared to control^41^. DBP exposure at 50 mg/kg/day did not alter histopathology in the small intestine^41^. In our study, we also observed significantly altered histopathological changes due to subacute DiNP exposure; however, these significant changes were observed in the colon at 0.02, 0.2, 2, and 200 mg/kg DiNP. Similar to DBP exposure at 50 mg/kg, DiNP exposure at 20 mg/kg did not alter histopathology in the colon. These data suggest that phthalates alter intestinal histopathology in a dose-dependent manner.

This study also investigated the impact of DiNP exposure on sex hormones in the colon. We measured the effects of DiNP exposure on hormone levels in the intestines because studies indicate that estradiol can be synthesized in gut-associated lymphoid tissues, such as the Peyer’s patches^18^. In a previous study conducted on a different set of mice using the same Illinois IACUC protocol, we investigated the impact of DiNP exposure on circulating sex hormones (i.e., hormones in the sera) and showed that DiNP exposure at 0.02, 0.1, and 200 mg/kg significantly decreased circulating testosterone levels and that DiNP exposure at 0.1 and 200 mg/kg significantly decreased circulating estradiol levels compared to control^9^. In the present study, we expanded these findings by showing that in the colon, DiNP exposure borderline decreased testosterone levels and significantly decreased estradiol levels in a dose-dependent manner compared to control. Thus, our previous study and the current study agree that DiNP exposure significantly decreases estradiol levels compared to control, suggesting that DiNP has antiestrogenic properties. Several studies have reported that DiNP is antiandrogenic at specific doses^9,42,43^. The present study shows that DiNP exposure causes a marginal decrease in testosterone levels in the colon compared to control, suggesting that DiNP also may have some antiandrogenic properties in the GIT. The DiNP-induced changes in hormone levels in the colon could stem from either DiNP-induced changes in the circulating levels of hormones that reach the colon or DiNP-induced changes in the local production of hormones by the colon. Future studies should examine these possibilities in detail.

Previous studies from our laboratory have shown that exposure to DBP alone^33^, a parent phthalate mixture of DEHP, DBP, diisobutyl phthalate (DiBP), benzyl butyl phthalate (BBzP), and DiNP^30^, and a phthalate metabolite mixture of monoethyl phthalate (MEP), mono(2-ethylhexyl) phthalate (MEHP), monobutyl phthalate (MBP), monoisobutyl phthalate (MiBP), monobenzyl phthalate (MBzP), and monoisononyl phthalate (MiBP)^44^ disrupt the expression of genes involved in apoptotic and cell cycle pathways in the ovary. Thus, we examined whether DiNP exposure alters the expression of mRNAs for various cell cycle regulators as well as cell proliferation and apoptosis in the colon. We found that although DiNP exposure significantly altered expression of apoptotic factors (*Aifm1* and *Bcl2l10*) compared to control, it did not significantly increase DNA fragmentation, an indicator of apoptosis, in the colon. Thus, it is possible that the colon is able to recover from changes in expression of apoptotic factors after subacute exposure to DiNP likely due to the quick turnover of colonocytes.

The critical role of the epithelial barrier in the immune regulation has been well documented in the colon^40^. Previous studies have shown that exposure to some phthalates increased intestinal inflammation and permeability^45^, but those studies did not examine the effects of DiNP on epithelial barrier function and immune function. Our current data indicate that DiNP exposure decreases the expression of *Zo-3*, which may explain why DiNP exposure causes changes in the gene expression and protein levels of various cytokines compared to control. The downregulation of tight junctions provides an ineffective barrier to luminal pathogens. Alternatively, DiNP exposure alters cytokine expression, which disrupts the intestinal tight junction barrier^46^. As a result, this would allow luminal antigens to penetrate intestinal tissues.

The present study examined gene expression and protein levels of various cytokines. DiNP exposure significantly increased *Tnf* expression at an environmentally relevant dose compared to control, but it did not significantly alter protein levels of TNF-$\alpha$ in all treatment groups compared to control. Although we did not observe significant changes at the protein level, it is interesting to note that the gene expression and protein level of this cytokine follow a similar dose–response curve. Although TNF-$\alpha$ levels were not significantly altered, TNF-$\alpha$ can still play a role in apoptosis and regulating other immune cells. It is unlikely that TNF-$\alpha$ plays a role in DiNP-induced apoptosis because TUNEL staining did not reveal significant cell death with DiNP treatment compared to control. It is possible that the interaction between TNF-$\alpha$ binding to TNF receptor type 1 (TNFR1) induces pro-inflammatory effects because we observed cellular infiltration in the colonic histology. We also examined sICAM-1 levels in the colon and observed a negative association between DiNP doses and sICAM-1 levels. sICAM-1 helps activate the immune system by promoting the interaction between macrophages and T-cells. These data suggest that DiNP-induced damage occurs by different mechanisms depending on the dose of DiNP.

To summarize these results, it is possible that when DiNP damages the colon, it induces local inflammation and apoptosis of colonic cells, and it also dowregulates expression of tight junctions. The downregulation of tight junctions could further exacerbate colonic inflammation because it allows microbes and microbial metabolites to pass the epithelial barrier. We observed changes in the cell cycle regulation, and this makes sense as cell cycle regulators are linked to cellular apoptosis and proliferation. The decrease in estradiol levels in the colon may also be contributing to the altered immune responses.

These results may have potential to be generalized to humans for several reasons. First, we exposed mice to environmentally relevant doses of phthalates that are in the range of exposure for humans of all ages. Second, we orally administered phthalates to mice in the way that humans are most commonly exposed to phthalates (i.e., ingestion). Third, mice have similar gastrointestinal anatomy and physiology to humans and have extensively been used as premier animal models for human gastrointestinal diseases. Humans and mice are similar in that they both have limited post-gastric fermentation because they are non-ruminants. Mice and humans also have a mouth, esophagus, stomach, small intestine, large intestine, and rectum. However, humans have a sacculated colon and fermentation largely takes place in the colon, whereas mice have a tubular colon and fermentation occurs in the cecum. The enlarged cecum in mice is much smaller in humans and is also known as the appendix. Thus, more research is needed to determine whether DiNP exposure affects immune responses, endocrine functions, and cell health in the colon of humans in an identical or similar manner in mice due to anatomical and physiological differences that exist in the GI tract of humans and mice.

## Conclusion

In summary, this study shows that acute exposure to DiNP increases colonic damage, decreases estradiol levels, downregulates expression of tight junctions, alters cytokine levels, and dysregulates expression of cell cycle regulators in the adult female mouse colon. Further studies are needed to understand the mechanisms underlying DiNP-induced effects on the colon. One possibility is that DiNP exposure alters the diversity of gut microbes, and this leads to changes in histopathology, cytokine level, immune function, tight junctions, and estradiol levels. Therefore, future studies should explore the impact of adult DiNP exposure on the gut microbiome in female mice.
